# Compound connection mechanism of Al_2_O_3_ ceramic and TC4 Ti alloy with different joining modes

**DOI:** 10.1038/s41598-021-00815-4

**Published:** 2021-10-28

**Authors:** Yan Zhang, YanKun Chen, JianPing Zhou, DaQian Sun, HongMei Li

**Affiliations:** 1 School of Mechanical Engineering, Xinjiang University, Wulumuqi, 830000 China; 2 Key Laboratory of Automobile Materials, School of Materials Science and Engineering, Jilin University, Changchun, 130022 China; 3 State Key Laboratory for Manufacturing Systems Engineering, Xian, 710000 China

**Keywords:** Engineering, Materials science

## Abstract

In this paper, laser welding-brazing of TC4 Titanium (Ti) alloy and Al_2_O_3_ ceramic dissimilar material was carried. The results showed that the Ti alloy and Al_2_O_3_ were joined by melting filler metal when the laser was concentrated in the Ti alloy side of the joint. The joint with one fusion weld and one brazed weld separated by remaining unmelted Ti alloy. Laser beam offset the Ti alloy 1.5 mm, Ti alloy would not be completely melted in joint. Through heat conduction, the filler metal melted occurred at the Ti-ceramic interface. A brazed weld was formed at the Ti-ceramic interface with the main microstructure of β-CuZn + Ti_2_Zn_3_, β-CuZn and Al_2_Cu + β-CuZn. The joint fractured at the brazed weld with the maximum tensile strength of 169 MPa.

## Introduction

Alumina (Al_2_O_3_) ceramics have excellent high temperature strength and corrosion and wear resistance, and are widely used in electronics, aerospace, nuclear, automotive and other industries^1–3^. Ti811 Ti alloy is an important part of the compressor blade of aviation engine, but its application is limited by its low surface hardness and poor wear resistance. A laser cladding composite coating was prepared on the Ti811 surface using the coaxial powder feeding method to improve the micro-hardness and wear resistance of the Ti811 alloy^4^. However, the inherent brittleness associated with Al_2_O_3_ ceramics limits their structural applications, particularly in the fabrication of complex geometries^5,6^. Therefore, the jointing of Al_2_O_3_ ceramics to themselves or to metals is usually preferred. Titanium-based (Ti) alloys have excellent high temperature strength, good creep properties and oxidation resistance and can be used in high temperature environments. The successful combination of Al_2_O_3_ ceramics and Ti alloys to prepare ceramic metal components can give full play to the advantages of the two materials and is widely used in vacuum pipes, energy converters, semiconductor devices, missiles, rockets and satellites. However, the main difficulty to realize the effective jointing between Al_2_O_3_ ceramics and Ti alloy lies in the great difference of their physical and chemical properties. The difference of physical properties is manifested in the mismatch of elastic modulus and linear expansion coefficient, which will lead to large residual stress at the interface after welding^7^. The difference in chemical properties is mainly due to the fact that the bonding form of the Al_2_O_3_ ceramic is mainly a covalent bond, and the Ti alloy is a metal bond.

Many jointing methods in the research of ceramic/metal connection technology has been reported, including brazing, diffusion welding, friction welding, local transition liquid phase jointing, ultrasonic welding, microwave welding, explosive welding, fusion welding, self propagating high temperature synthesis welding and hot pressing reaction sintering connection^8–13^. At present, brazing is one of the most effective ways to joint metal and ceramic materials. However, brazing of ceramics presents difficulties because conventional filler metals do not easily wet alumina. Recently, several welding techniques have been documented^14^, such as vacuum brazing^15^, diffusion bonding^16^, and partial transient liquid phase (PTLP) brazing^17^. Among these methods, active brazing is a relatively simple and reliable technology for bonding Al_2_O_3_ ceramics due to the improved wettability and joining quality by active elements in braze alloys^18^. In the brazing joint of metal and ceramic, active elements can be added to the brazing filler metal to improve the wetting ability of the brazing filler metal to the ceramic joint interface. The most commonly used active element in the solder is Ti, followed by Zr, V, Cr, etc.^19^. However, the conditions of use may make a particular process inappropriate. The vacuum brazing process generally takes a long time to implement. In addition, the required joint geometry may make brazing difficult to apply. In fact, the butt welding of Al_2_O_3_ and Ti alloy is technically convenient^20^. As an efficient and flexible non-contact welding technology, laser welding has made great achievements in the jointing of welding refractory materials and dissimilar metals.

Based on the above analysis, two welding mechanisms (fusion welding and brazing) are combined to avoid melting and liquid mixing of the base metals during welding. Using laser as welding heat source and Cu-based fillers as interlayer material. The welding process was set up to ensure that the Ti alloy was partly melted as expected from previous work^21^. The melted Ti alloy formed a fusion weld. Meanwhile, a brazed weld was formed at the interface between unmelted Ti alloy and Al_2_O_3_. In this way, a peculiar joint was acquired. So far, this kind of joint has been rarely reported.

## Experimental

**Figure 1:**
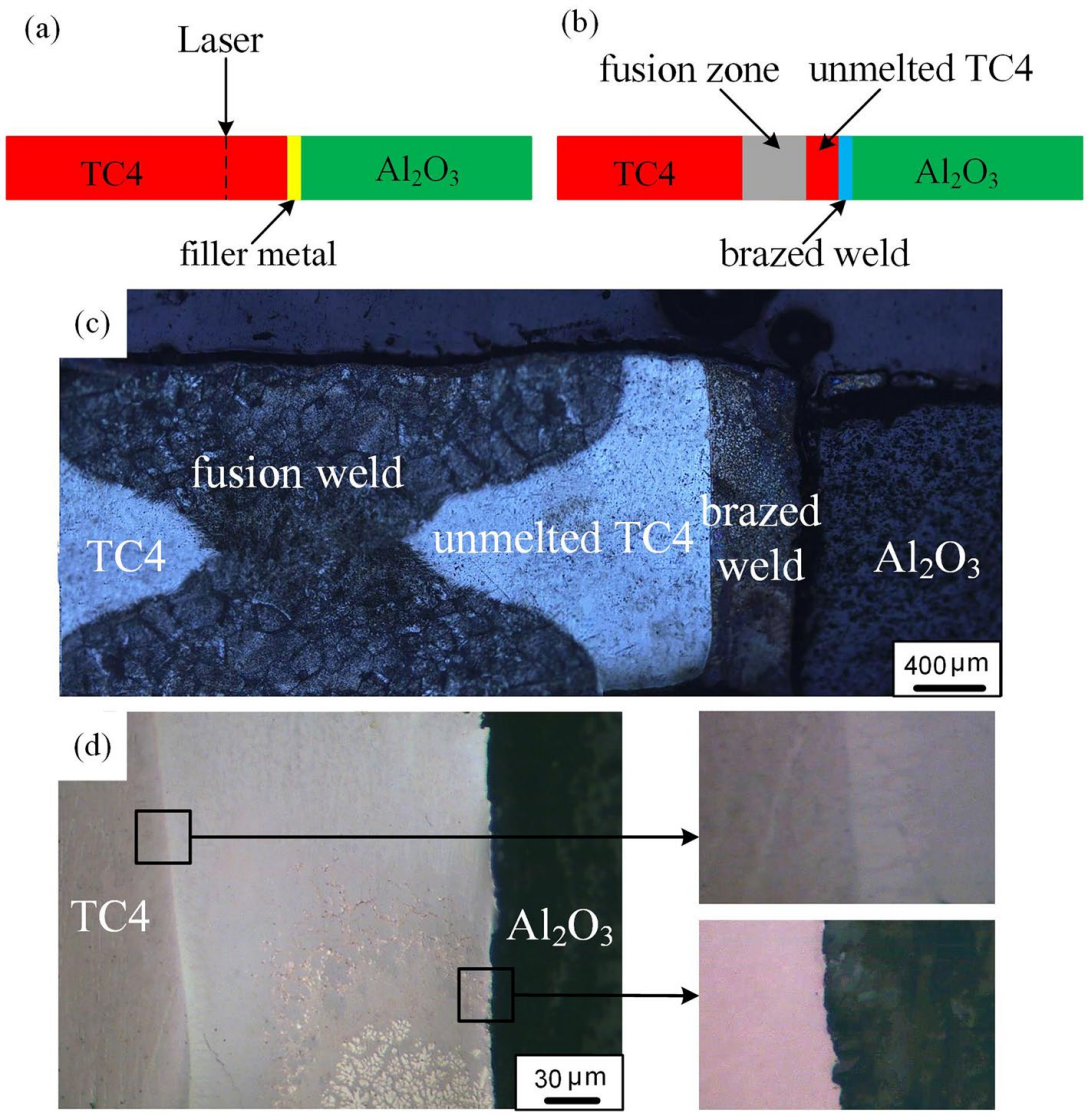
**(a)** Schematic diagram of the welding process; **(b)** design scheme of one pass laser welding joint; **(c)** optical image of the cross section of the joint; **(d)** optical image before corrosion of the Ti alloy-Al_2_O_3_ alloy interface.

The TC4 alloy plates (88 wt.% Ti, 6.06 wt.% Al and 4.03 wt.% V) and Al_2_O_3_ ceramic plates (Al_2_O_3_ 96%, SiO_2_ 2%, B_2_O_3_ 0.5% and C_3_H_5_NO 0.8%) were 1 mm thick, the sample plates size were 40 mm × 20 mm × 1 mm. The filler metal was 0.2 mm Cu-based filler (61.2 wt.% Cu, 37.2 wt.% Zn, 0.28 wt.% Si, and 0.89 wt.% Sn). The specimen was mechanically and chemically cleaned before welding. The gap between the Ti alloy and the ceramic was important for adequate heat transfer and prevention of pore formation. In order to minimize the gap between the edges, the test pieces were clamped to each other. A continuous laser with an average power of 1.20 KW, a wavelength of 1080 nm and a spot diameter of 0.1 mm was used. The welding process is shown in the Fig. 1a,b. In order to ensure that the Ti alloy was not completely melted, the laser beam was focused on the Ti alloy plate with a distance of 1.5 mm from the Ti-filler metal interface. The welding parameters were: laser beam power of 420 W, defocusing distance of + 5 mm, welding speed of 550 mm/min. After welding, the surface of the joint was polished to a smooth state to perform corrosion and optical microscope tests. The specimens and tensile specimens were machined by wire electrical discharge machining. Tensile strength of the joints was measured by using universal testing machine (MTS Insight 10 kN) with cross head speed of 0.2 mm/min. Argon gas with the purity of 99.99% was applied as a shielding gas with total flow of 20 L/min at top of the joint.

## Results and discussion

The optical microscope (OM) image of the cross section of the joint is shown in the Fig. 1c. It could be seen that the joint was divided into three parts: the fusion weld formed at the Ti alloy side, unmelted Ti alloy and the brazed weld formed at the Ti-Al_2_O_3_ interface. The average width of fusion weld, unmelted Ti alloy and brazed weld was 1.2 mm, 0.55 mm and 0.25 mm, respectively. Local heating on the Ti alloy side produced an uneven volume expansion and thermal stress, which helped to obtain a close joining between the Ti alloy, filler metal and Al_2_O_3_ interface. The high temperature and the close contact at the Ti-Al_2_O_3_ interface provided favorable conditions for atomic diffusion, which leads the brazing process at the Ti-Al_2_O_3_ interface very well. The microstructure of fusion weld was quite different from that of braze weld, and the brazed weld became black after corrosion. Figure 1d shows the OM image of the brazed weld before corrosion. It was not found the pores, macro-cracks and other defects. In addition, the eutectic joining between Ti and filler was realized by heat conduction, and the Cu-based filler has good ductility, it was beneficial to alleviate and adjust the thermal stress of the Ti-Al_2_O_3_ joint, which was beneficial to improve the mechanical properties of the joint.

In order to obtain a good joint, it was especially important to precisely control the position of the laser spot. When the laser spot was away from the Ti alloy interface, it could not be brazed at the Ti-Al_2_O_3_ interface. When the laser spot was close to the Ti alloy interface, the Ti alloy and Cu filler began to melt in the braze weld, and the content of the brittle intermetallic compound was greatly increased, and the joining of the Ti/Al_2_O_3_ dissimilar material could not be achieved.

**Figure 2:**
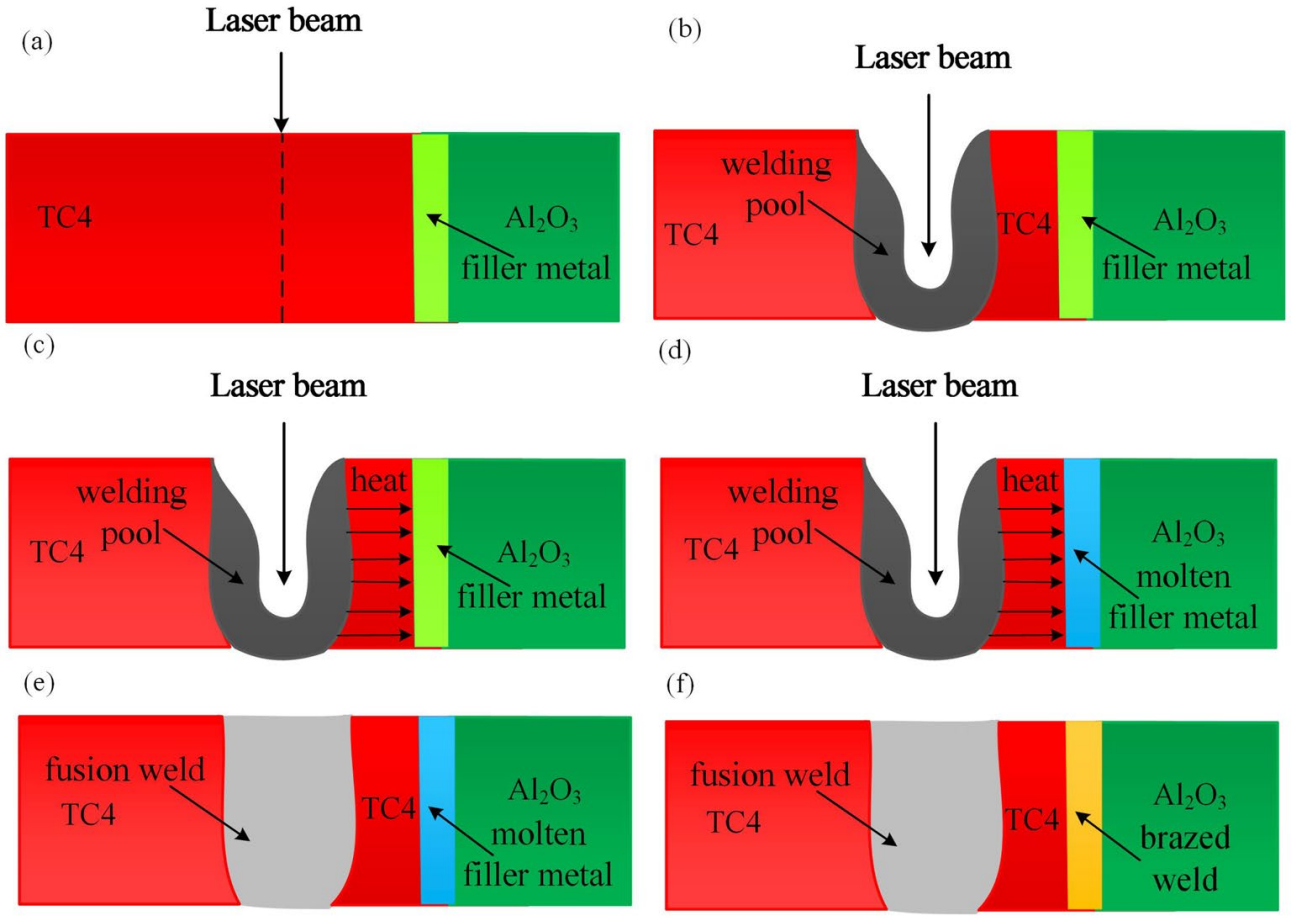
**(a)** Laser beam was focused on the Ti alloy plate; **(b)** formation of welding pool on Ti alloy side; **(c)** heat was transferred from welding pool to the Ti alloy-Al_2_O_3_ interface; **(d)** atomic interdiffusion at Ti alloy-Al_2_O_3_ interface; **(e)** formation of fusion zone on Ti alloy side; **(f)** formation of brazed weld on Ti alloy-Al_2_O_3_ interface.

Figure 2 shows the physical model formed of the joint. When the laser beam was fixed to the side of the Ti alloy plate at a certain distance from the interface of the Ti alloy, a molten pool with small holes was generated inside the Ti alloy plate, thereby absorbing a large amount of heat, as seen in Fig. 2a,b. Due to the high absorption of the laser in the small holes, the Ti alloy at the boundary of the small holes was sharply melted, but the Ti alloy side was not completely melted^22^. The temperature of the unmelted Ti alloy increased rapidly with the increase of the heat of the small hole boundary, and was transmitted to the Al_2_O_3_ side, as seen in Fig. 2c. Therefore, the unmelted Ti alloy had a sufficiently high temperature to promote melting of the filler metal at the Ti-Al_2_O_3_ interface, as seen in Fig. 2d. The solidification of the weld pool and the liquid filler metal in the joint could start from the Ti alloy side, resulting in a composite joint between the fusion weld and the braze weld, as seen in Fig. 2e,f.

**Figure 3:**
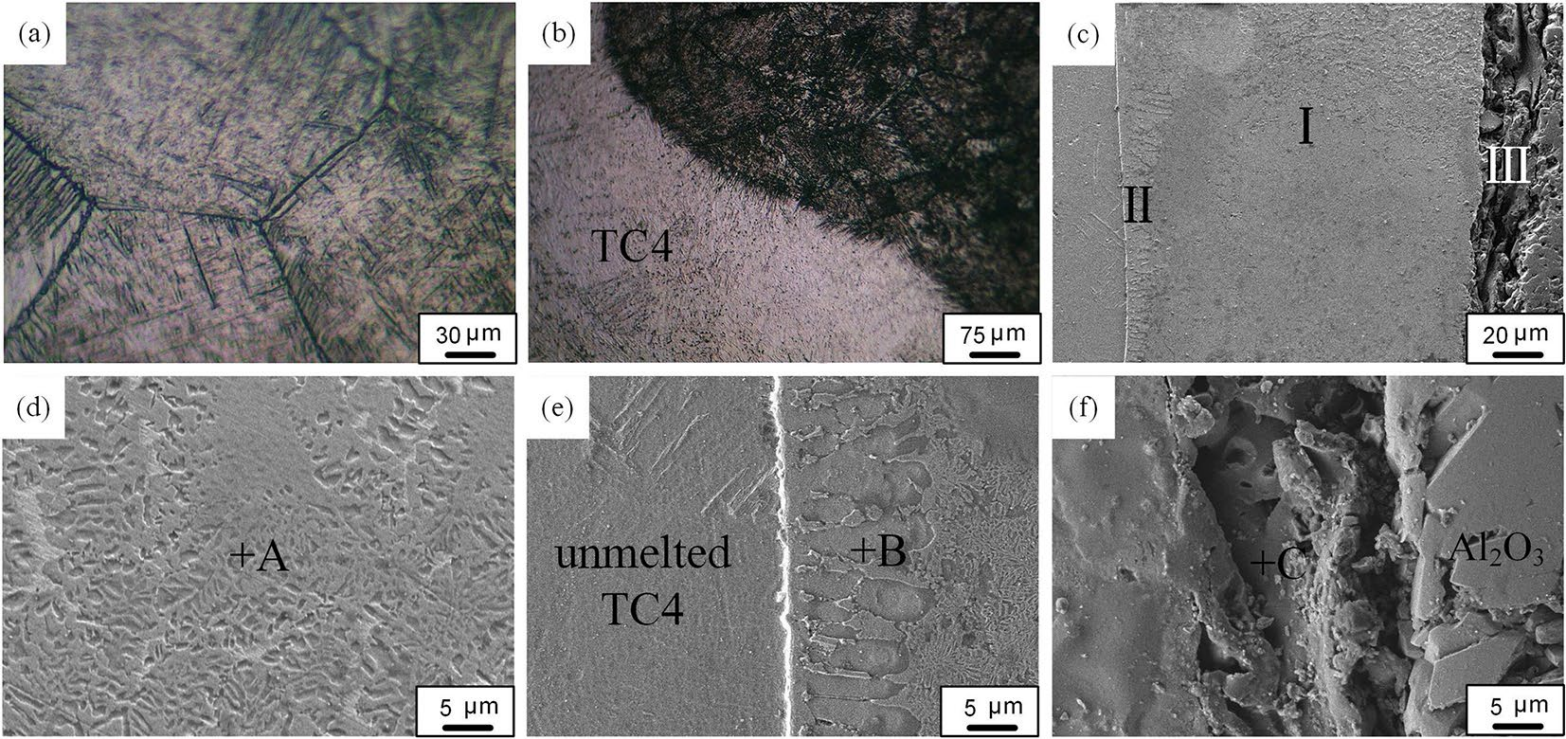
**(a)** Optical image of fusion zone; **(b)** optical image of fusion line; **(c)** SEM image of the brazed weld; **(d)** SEM image of the zone I in **(c)**; **(e)** SEM image of the zone II in **(c)**; **(f)** SEM image of the zone III in **(c)**.

The OM image of the fusion weld is shown in Fig. 3a,b, and there were no defects in it. The fusion weld mainly consists of acicular α' martensite. The SEM image of the brazed weld is shown in the Fig. 3c. The results showed that the brazed weld was a layered structure, which could be divided into three zones I, II and III according to the shape and color, as shown in the Fig. 3c. Figure 3d–f correspond to the three zones in Fig. 3c, respectively. The composition of each reaction zone (indicated by the letters A-C in Fig. 3) was investigated by SEM–EDS, and the composition of the reaction product was measured. The results were shown in in Table 1. According to the previous analysis, the microstructure of the brazed weld was mainly composed of molten filler metal. The composition of A point in the Zone I was consistent with the filler metal. According to the results of the energy spectrum analysis and the Cu–Zn phase diagram, the main microstructure of the zone I was determined to be β-CuZn phase. When the laser beam was focused on one side of the Ti alloy, elemental diffusion between the matrix material and the filler occurred immediately, resulting into their composition to deviate from the original composition. Therefore, liquid phase formation and elemental diffusion occurred simultaneously. As the solid phase dissolved into the liquid phase, impurities in the liquid phase diffused into the Al_2_O_3_ and Ti alloy to form a solid phase reaction layer, which existed only in a small area of the interface. As shown in the Fig. 3e,f, zones II and III were reaction layers formed by elemental diffusion. According to the Cu–Zn–Ti phase diagram^17,23^, it was determined that the microstructure of the zone II and the zone III was β-CuZn + Ti_2_Zn_3_ phase, Al_2_Cu + β-CuZn phase, respectively. Therefore, the main microstructures of brazed weld were β-CuZn + Ti_2_Zn_3_, β-CuZn and Al_2_Cu + β-CuZn. Oxygen was mainly distributed in the reaction layer on the ceramic side, and no TiOx-type phase was detected. Titanium was not detected in this compound, and the ceramic is melted and wetted by thermally conductive solder, but the filler itself does not contain titanium, and the titanium in the brazing weld comes from the Ti alloy.

**Table 1:** The chemical composition of each phase (wt.%).

Region	Composition%							Potential phases
	Ti	Al	Cu	Zn	Si	O	V	
A			57.3	39.6				β-CuZn
B	40.6		36.7	20.5				β-CuZn + Ti_2_Zn_3_
C		50.1	23.6	15.2		10.6		Al_2_Cu + β-CuZn

**Figure 4:**
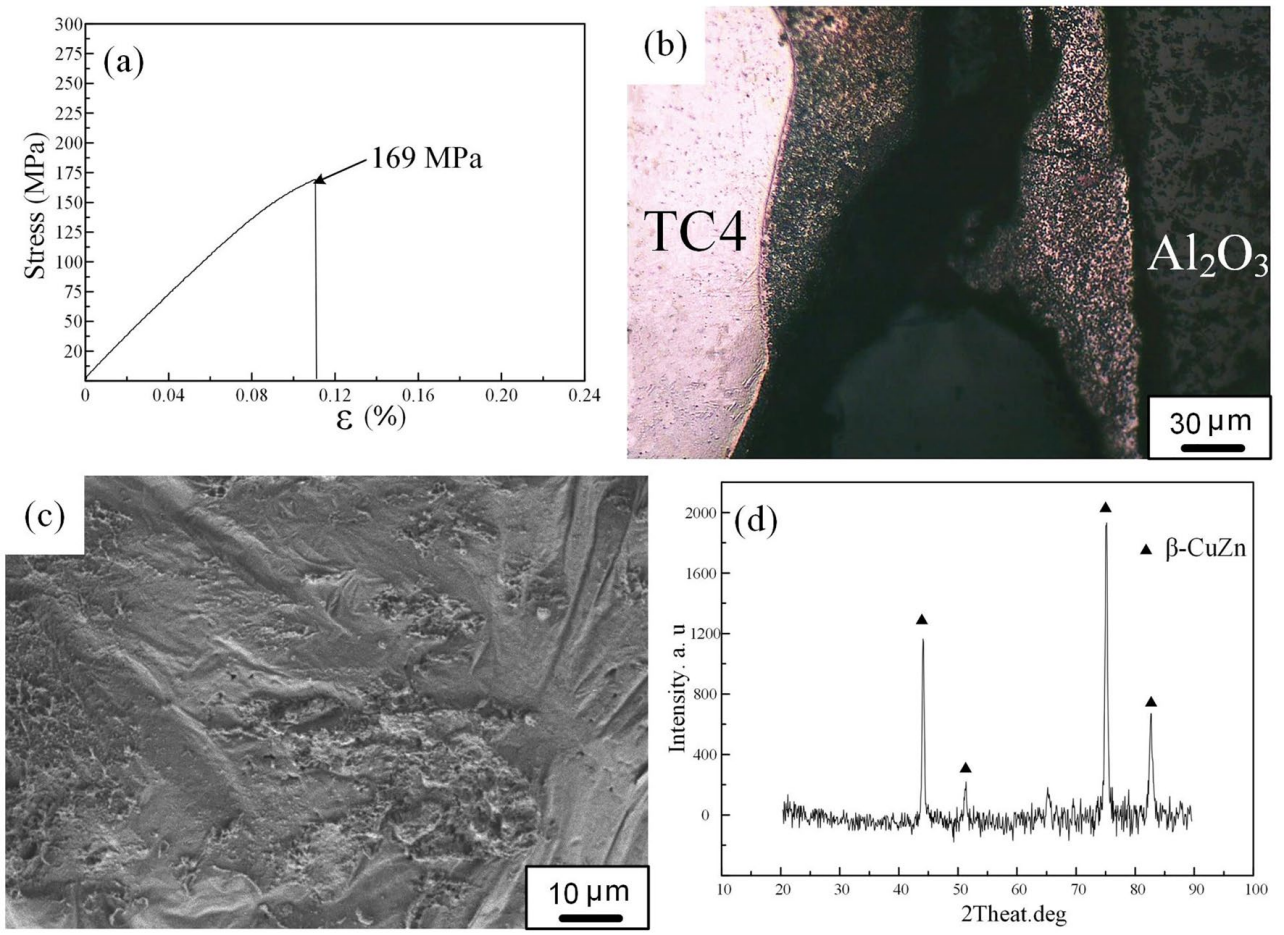
**(a)** Tensile test curve; **(b)** fracture location; **(c)** SEM image of fracture surface; **(d)** XRD analysis results of fracture surface.

Prepared three tensile specimens for each weld. The maximum tensile strength of the joint was about 169 MPa (Fig. 4a). There are minor differences in the processing of each sample, resulting in slightly different tensile strength. So the average tensile strength of the joint was about 147 MPa. The joint fractured in zone I of the brazed weld during tensile tests (Fig. 4b), which indicates that filler has good wettability on base materials. Figure 4c shows fracture surface of the joint exhibiting typical brittle characteristics. Moreover, as shown in Fig. 4d, XRD analyses of fracture surface detected β-CuZn phase. This confirmed the presence of β-CuZn phase at fracture surfaces. It should be noted that there was no Ti-Fe intermetallics in the brazed weld. The brazed weld became the weak zone of the joint, which led to the failure in the tensile test.

## Conclusions

Due to the presence of unmelted Ti alloy, the formation of intermetallic compounds was not found in the fusion weld and brazed weld, which improved the mechanical properties of the joints. A brazed weld was formed at the Ti alloy-Al_2_O_3_ interface with the microstructure of β-CuZn + Ti_2_Zn_3_, β-CuZn and Al_2_Cu + β-CuZn. A great amount of atomic diffusion occurs at the Ti alloy-Al_2_O_3_ interface during welding, and the thickness of diffusion weld can reach tens of micrometres. The tensile resistance of the joint was determined by brazed weld. The maximum tensile strength of joint was 169 MPa.
